# Prevalence and associated factors of pediatric hypertension in Jazan region, south of the Kingdom of Saudi Arabia. A pilot cross-sectional study

**DOI:** 10.1371/journal.pone.0287698

**Published:** 2023-07-10

**Authors:** Maged El-Setouhy, Abdulrahman M. Safhi, Musab Y. Dallak, Ahmed Y. Ayoub, Osama A. H. Suwaid, Ahmed K. Moafa, Alhassan M. Al-ahmed, Mohammad Zaino, Ahmed Al Sayed

**Affiliations:** 1 Department of Family and Community Medicine, Faculty of Medicine, Jazan University, Jazan, Kingdom of Saudi Arabia; 2 Department of Community Environmental and Occupational Medicine, Faculty of Medicine, Ain Shams University, Cairo, Egypt; 3 Department of Emergency Medicine, Faculty of Medicine, Maryland University, Baltimore, MD, United States of America; 4 Medical Laboratory Technology Department, Faculty of Applied Medical Science, Jazan University, Jazan, Kingdom of Saudi Arabia; 5 Department of Medicine, Faculty of Medicine, Jazan University, Jazan, Kingdom of Saudi Arabia; North South University, BANGLADESH

## Abstract

Hypertension (HTN) is a primary global health concern. Moreover, according to the 2010 Global Burden of Disease, hypertension accounted for roughly a quarter of cardiovascular disease fatalities and 1.9 percent of all deaths in Saudi Arabia in 2010. Also, hypertension is a significant risk factor for cardiovascular disease, morbidity, and mortality. However, assessing blood pressure (BP) and preventing hypertension among children and adolescents has become a global priority. This study aims to determine the prevalence of hypertension among children in the Jazan region of Saudi Arabia. Also, to determine the common risk factors associated with pediatric hypertension. We conducted this cross-sectional study among boys and girls aged 6–14 years visiting Al-Rashid Mall, one of the two main malls in Jazan city, the capital of Jazan region, Saudi Arabia, between November 2021 and January 2022. We included children willing to participate in the study after obtaining their parents’ consent and children’s assent. We used a standardized questionnaire to interview the parents to collect the children’s data. We also measured the children’s resting BP. Then we classified the measurements according to the updated International Pediatric Hypertension Association (IPHA) chart. We also measured the height and weight of the children and calculated their BMI. We used SPSS version 25 for the data entry and analysis. Our results showed that the prevalence of hypertension and prehypertension was insignificantly higher in females (11.84% and 12.65%) compared to males (11.52% and 11.52%), respectively. Our participants’ main associated factors with prehypertension and hypertension were overweight, obesity, and family income. Pediatric hypertension and prehypertension were highly prevalent in Jazan region. Therefore, being overweight and obese should be considered risk factors for pediatric hypertension. Our study emphasizes the need for early intervention to prevent pediatric HTN, particularly among overweight and obese children.

## Introduction

High blood pressure (hypertension) continues to be a global health problem. It was responsible for a yearly 10.2 million deaths and 208 million years of modified life for disability [1]. In 2021, the Saudi Minister of health MOH declared that two out of five adults in the middle east are suffering from hypertension [2]. However, earlier Saudi studies showed that hypertension (HTN) prevalence among adults was around 49% [3]. This high prevalence of hypertension in adolescents and adults would be associated with an increase in pediatric hypertension [4].

Moreover, persistent childhood hypertension leads to adulthood hypertension and other chronic diseases [5, 6]. That is why assessing blood pressure (BP), detecting HTN, and preventing it among children and adolescents became a global priority [7, 8]. Pediatric HTN significantly contributes to cardiovascular diseases (CVD) and end-stage renal diseases. Therefore, early detection and treatment of pediatric hypertension would substantially impact children’s lives [4, 9–12]. It can lead to complications such as heart failure, left ventricular hypertrophy, and stroke [5, 12, 13].

On the other hand, narrowing the blood vessel within the kidney or a blood clot in an artery (renal artery stenosis- thrombosis) within the kidney can cause high blood pressure in children. Children with kidney problems such as nephrotic syndrome and acute and/or chronic renal failure are also at higher risk of high blood pressure. Other disorders, such as thyroid problems, neurofibromatosis, and tuberous sclerosis, increase the risk of HTN [14, 15]. However, risk factors for developing childhood hypertension are still not well defined. Some risk factors were age, gender, body size, socioeconomic status, family history of hypertension, changes in dietary habits, sedentary lifestyle, dietary sodium intake, and increased stress [16–22]. Obese children are always at higher risk of being hypertensive [23–25]. The prevalence of HTN among children in western studies ranged from 7 to 19% [26–28]. The prevalence of pediatric hypertension in Saudi Arabia increased significantly and is considered a pediatric health problem. An earlier (more than ten years) national study included 16,226 Saudi children and adolescents aged birth to 18. The result showed that boys’ and girls’ blood pressure increases steadily with age. The average annual increase in systolic blood pressure (SBP) for boys was 1.66 mm Hg, whereas, for girls, it was 1.44 mm Hg. The diastolic blood pressure(DBP) increased annually with an average of 0.83 mm Hg for boys and 0.77 mm Hg for girls increased substantially in boys at the age of 18 years, while DBP increased dramatically in boys at the age of 18 years [29–31]. The literature review showed a graded relationship between adulthood and childhood hypertension [32, 33]. However, no studies were created on pediatric hypertension in Jazan region. That is why we created this study to investigate hypertension prevalence and determinants among children aged 6–14 years in Jazan Region, Kingdom of Saudi Arabia.

## Materials and methods

Jazan region is the south border of the Kingdom of Saudi Arabia. Most of the regions are rural areas that have shown some urbanization recently. It is a unique place where medical research was only considered when Jazan University was established in 2006 to start medical education and research.

We created this analytical cross-sectional study on a convenient sample of 601 children between the ages of 6–14 who were visiting Al-Rashid Mall (one of the two main malls in Jazan city, the capital of Jazan region). We selected Al-Rashid Mall as it is the only mall with cinemas that were new to the region then, so most of the region’s children were visiting it. After getting their guardians’ consent, we included all children willing to participate in the study. We created this study between November 2021 and February 2022 to include the mid-year holiday, when most of the children in Jazan region were visiting the malls with their families. We also selected the weekend days to catch most of the children from different governorates in the region. All children visiting the mall were invited to participate. However, only 601 children’s guardians accepted for their kids to participate in the study. For data collection, we used a pre-prepared questionnaire to collect demographic data as well as data on the risk factors of hypertension. Before using this questionnaire was validated professionally by a group of pediatricians, epidemiologists, and cardiologists (5 consultants) from Jazan University and Jazan Ministry of Health. Then we created a pilot study on 25 children and their guardians, not included in the study, to evaluate the understandability and reliability of the questionnaire. We also measured the BP of each child using a validated electronic sphygmomanometer with a pediatric cuff. The BP was measured on the right arm after a rest period of 5 minutes, with the right arm supported and the cubital fossa at the heart level while the children were sitting. We obtained two readings at an interval of 2–3 min. We recorded the average of those readings. When the BP measurements were higher than the 90th percentile for age, gender, and height, the measurement was repeated after five more minutes, and we considered the average [34]. For evaluation, we used the updated International Pediatric Hypertension Association (IPHA) chart to calculate the BP threshold values for each gender and 5 cm height group [35]. The IPHA chart classifies hypertension in children and adolescents as prehypertension (BP higher than the 90th percentile but less than the 95th percentile), stage I HTN (> 95th percentile but <99th percentile), and stage II HTN (> 99th percentile). We also measured the weight and height of the children using a validated scale. Then the children’s BMI was calculated and expressed as a percentile interpreted using the body mass index-for-age percentiles chart [34]. Because of the change in weight and height during growth and development, the children’s Body Mass Index (BMI) was interpreted concerning other children of the same sex and age [36].

### Ethical consideration

The study proposal was reviewed and accepted by the Jazan University Research Ethics Committee (REC-43/06/124.). In addition, we collected written informed consent from the children’s guardians and verbal assent from the children. All collected data were kept confidential.

### Statistical analysis

We used SPSS (Statistical Product and Service Solutions) version 25 for data entry and analysis. Chi square test was used to test the association between pediatric blood pressure with the categories (Normal, prehypertension, and hypertension) and sociodemographic data. In case of significant results, Z-Test was used to determine which categories were responsible for that significant association.

To analyze the relationship between the dependent variable "Hypertension" and its three levels, normal, prehypertension, and hypertension, we used the cumulative odds ordinal logistic regression with proportional odds, using cumulative categories.

We then used the tolerance measures to check the multi-collinearity, which strengthens the linear relationships among the independent variables included in the model. All tolerance values were above 0.6, indicating no sign of multi-collinearity existence [37]. So our model is considered statistically ‘stable’.

## Results and discussion

Table 1 shows that the 601 children in this study were 356 (59.2%) males and 245 (40.8%) females. The prevalence of hypertension and prehypertension were insignificantly higher among females than males. The highest prevalence of hypertension was among the 12–14 age group. Regarding BMI, hypertension was significantly higher among overweight and obese children (38.23% and 45.76%, respectively). Moreover, prehypertension was significantly higher among the obese group (25.42%). The presence of chronic diseases and the parents’ consanguinity were not significantly related to our participants’ prevalence of hypertension or prehypertension.

**Table 1:** Relation between blood pressure classification and participant’s characteristics.

Variables	Blood pressure			P
	Hypertension	Prehypertension	Normal	
	N (%)	N (%)	N (%)	
Gender				
Male (n = 356)	41 (11.52)	41 (11.52)	274(76.96)	0.901
Female (n = 245)	29 (11.84)	31 (12.65)	185(75.51)	
Age				
6–8 (n = 203)	26 (11.81)	24(11.82)	153(75.37)	0.735
9–11 (n = 234)	22 (9.4)	26 (11.11)	186(79.49)	
12–14 (n = 164)	22(13.415)	22(13.415)	120(73.21)	
BMI				
Underweight (n = 156)	30 (19.23)	18 (11.54)	108 (69.23)	0.000
Normal body weight (n = 437)	87 (19.91)	74 (16.93)	276 (63.16)*	
Overweight (n = 102)	39 (38.23)*	22 (21.57)	41(40.19)	
Obese (n = 118)	54 (45.76)*	30 (25.42)*	34 (28.81)	
Consanguinity of the parents				
Yes (n = 268)	67 (12.2)	67 (12.2)	414 (75.6)	0.784
No (n = 333)	3 (5.6)	5 (9.4)	45 (85)	
Have Brothers & sisters				
Yes (n = 548)	67 (12.2)	67 (12.2)	414 (75.6)	0.265
No (n = 53)	3 (5.6)	5 (9.4)	45 (85)	
Child Chronic Diseases				
Yes (n = 31)	2 (6.45)	4 (12.91)	25 (80.64)	0.651
No (n = 570)	68 (12)	68 (12)	434 (76)	

* Indicates the cell proportion responsible for the significant result of Chi-square test after adjusting with Z-test.

Table 2 demonstrates the relationship between blood pressure and demographic factors. The father’s occupation, being an employee, was the only significant factor associated with hypertension among the studied children.

**Table 2:** Socio-demographic data of the participants concerning blood pressure classification.

Variables	Blood pressure			P
	Hypertension	Prehypertension	Normal	
	N (%)	N (%)	N (%)	
Residence				
Urban (n = 362)	52 (14.36)	42 (11.61)	268 (74.03)	0.071
Rural (n = 245)	17 (7.87)	29 (13.43)	170 (78.70)	
Mountain (n = 23)	1 (4.35)	1 (4.35)	21 (91.30)	
Family history of hypertension				
Yes (n = 147)	20 (13.60)	20 (13.60)	107(72.81)	0.497
No (n = 454)	50 (11.013)	52 (11.453)	352 (77.534)	
Father occupation				
Employee (n = 448)	68 (12.41)*	62 (11.31)	418 (76.28)	0.014^f^
Unemployed (n = 37)	2 (5.41)	7 (18.92)	28 (75.67)	
Self-employed (n = 16)	0 (0)	3 (18.75)	13 (81.25)	
Father education				
Illiterate (n = 9)	0 (0)	0 (0)	9 (100)	0.081^f^
Primary school (n = 19)	5 (26.31)	2 (10.53)	12 (63.16)	
Intermediate school (n = 33)	4 (12.1)	4 (12.1)	25 (75.8)	
Secondary school (n = 184)	16 (8.7)	14 (7.6)	154 (83.7)	
Bachelor (n = 310)	38 (12.26)	44 (14.19)	228(73.55)	
Master or doctorate (n = 46)	7 (15.2)	8 (17.4)	31 (67.4)	
Mother occupation				
Employee (n = 222)	28 (12.6)	29 (13.1)	165 (74.3)	0.736
Unemployed (n = 373)	42 (11.26)	43 (11.52)	288 (77.21)	
Self-employed (n = 6)	0	0	6 (100)	
Mother education				
Illiterate (n = 33)	1 (3)	4 (12.1)	28 (84.9)	0.066^f^
Primary school (n = 20)	6 (30)	0 (0)	14 (70)	
Intermediate school (n = 34)	1 (2.94)	4 (11.76)	29 (85.3)	
Secondary school (n = 188)	20 (10.64)	20 (10.64)	148 (78.72)	
Bachelor (n = 317)	40 (12.62)	42 (13.25)	235 (74.15)	
Master or doctorate (n = 9)	2 (22.2)	2 (22.2)	5 (55.6)	
Family income				
<5000 SR (n = 35)	3 (8.57)	4 (11.43)	28 (80)	0.792
5000–9999 SR (n = 152)	16 (10.52)	17 (11.18)	119 (78.30)	
10,000–14.9999 SR (n = 229)	33 (14.41)	27 (11.80)	169 (73.79)	
>15,000 SR (n = 185)	18 (9.73)	24 (12.97)	143 (77.29)	

* Indicates the cell proportion responsible for the significant result of Chi-square test after adjusting with Z-test.

^f^ Shows the Fisher exact test results in case there was a cell frequency less than 5%.

SR = Saudi Riyals (U$1 = 3.76SR).

Table 3 shows that the odds of overweight children having hypertension was 2.22 (95% CI, 1.24 to 3.90) times that of normal weight children, a statistically significant effect, Wald χ2(1) = 7.142, p = .008. On the other hand, obese children were 3.57 (95% CI, 2.06 to 6.2) times more likely to have hypertension compared to those with normal weight, a statistically significant effect, Wald χ2(1) = 20.485, p < .0001.

**Table 3:** Predictors of children hypertension in Jazan region*.

Predictor Variables	Estimate	Std. Error	Wald	df	Sig	expb	95% CI	
BMI = 1	-0.327	0.294	1.236	1	0.266	0.72	0.4	1.3
BMI = 2	0.792	0.296	7.142	1	0.008	2.21	1.24	3.9
BMI = 3	1.271	0.281	20.485	1	0.000	3.57	2.06	6.2
BMI = 4, ref. group								
F_income = 1	0.162	0.573	0.081	1	0.777	1.18	0.38	3.6
F_income = 2	0.292	0.314	0.869	1	0.351	1.34	0.72	2.5
F_income = 3	0.518	0.26	3.975	1	0.046	1.68	1.01	2.8
F_income = 4, ref. group								

BMI:1 = underweight, 2 = overweight, 3 = obese, and 4 = normal weight.

F_income: Family income with 1 = less than 5000 SR, 2 = 5000 to 1000 SR, 3 = 10000 to 150000 SR, and 4 = more than 15000 SR.

*What is shown are only the significant predictors controlled for the independent variables residence, family history, father education, father occupation, mother education, and mother occupation. The level of significance was taken at 0.5%

The table results also indicate that for families with income between 10000 and 15000 SR, the odds of having an adult with hypertension is 1.68 (95 CI, 1.01 to 2.8) times that of income more than 15000 SR. This effect was significant, Wald χ2(1) = 3.957, p = 0.046.

Cardiovascular diseases are the leading causes of death worldwide. Moreover, hypertension is a significant risk factor for nearly all cardiovascular diseases [3]. It is believed that the persistent rise of BP during childhood usually leads to adulthood hypertension. That is why pediatric BP measurement has become of global importance. However, most pediatricians in the Middle East still do not consider measuring BP during the routine examination of children [38]. Literature is scarce on pediatric hypertension in our area. Moreover, we did not find any literature about the prevalence of pediatric hypertension in Jazan region. The prevalence of pediatric hypertension was reported to be 1–5% worldwide, rising to 10% in some areas. Prehypertension prevalence of was as high as 30% in some areas of the world [3]. However, the prevalence of hypertension and prehypertension was 3.4% and 3.6% among American children aged 3−18 years [39]. In Saudi Arabia, a study created in the pediatric clinic at King Abdulaziz University Hospital in Jeddah among children aged 6 to 15 years showed that the prevalence of hypertension and prehypertension in males was 14.4% and 6.5%, respectively. While in females, the prevalence of hypertension and prehypertension was 16.3% and 5.2%, respectively [31]. The number of children participating in this study was 369, 216 boys and 153 girls. In our research, we had more participants (601 children); 356 (59.2%) were males, and 245 (40.8%) were females. The prevalence was slightly higher among females than males, as shown in Table 1. Although our results showed a higher prevalence than the Jeddah study, we did not detect a significant gender difference regarding the prevalence of pediatric hypertension, which agrees with that study [31]. This would be explained by the different settings used to recruit the children as we used the mall sitting, and they used the clinic sitting with fewer participants. The highest prevalence of hypertension in our study was in the group 12–14 years old. Nevertheless, there were no statistical differences among age groups. Others also detected these findings [40].

Regarding BMI, our findings showed that hypertension and prehypertension were more common among obese and overweight children. Our results are consistent with earlier global and Saudi studies [31, 41–43]. Moreover, obesity was the leading risk factor for children’s hypertension in Jazan region (Tables 1 and 3). Another study published in 2009 observed that obese children were 5 to 6 times more at risk of getting high BP than non-obese children [44]. Obese children would sometimes be bullied for their obesity, which would also lead to lowering their self-esteem, feelings of worry and shyness, and putting them under stress. All these factors usually lead to chronic medical disorders, including hypertension [25, 45–49].

Regarding the father’s occupation, our findings showed that children whose fathers were employees were more likely to develop hypertension than others. Our results revealed that 68 (12.41%) children with employed fathers had hypertension. Employed fathers would be less capable of monitoring their child’s health status. Also, we understand that employees are usually more educated than the unemployed and self-employed, although they would be more aware of the risks of such diseases. One of the main variables we were interested in asking about is the total family income. Our participants’ higher prevalence of hypertension was among children with a family income of 10,000–14.9999 SR (around U$3,000–4,000). This result would be explained by the fact that this is the income range of most middle to early high-level employees in the area, as the father’s occupation was masked by the income (Table 3). Another recent study supported our findings as well [50].

Regarding residency, although urban resident children experienced nearly double the prevalence of hypertension (14.36%) than those who resided in rural areas (7.87%), the differences were not statistically significant (Table 1). According to another study, there were significant discrepancies between urban and rural people, particularly in systolic blood pressure. The systolic blood pressure of urban adolescents was higher than that of non-urban adolescents. Rural boys had significantly higher diastolic blood pressure compared to urban boys. Even after adjusting age and height, the inequalities remained [51, 52]. Our study was not consistent with these findings. Other variables show no statistical differences.

When we did a logistic regression (Table 3), all factors showed insignificant association with children’s hypertension except obesity and family income, which supports other studies’ findings in this area.

## Conclusions

Pediatric hypertension is prevalent in Jazan region, especially among obese and overweight children with moderate family income. So, measuring blood pressure is a necessity during pediatric check-ups. In addition, awareness campaigns should be held to raise public awareness of this growing health problem and the importance of lifestyle modification for obese and overweight children to prevent the subsequent development of hypertension. We suggest measuring the child’s physical activity and fat diet intake in future research.

### Limitations

We used a convenient sample in this study to find out and get some understanding of the prevalence of pediatric hypertension. However, this was the best method to reach most Jazan children, and we believe that our sample represents most of the children in the region. The response rate was around 50%. School sittings with random sampling are recommended for further studies to get better representation but need more funds and personnel.

## Supporting information

S1 File (PDF)

S2 File (DOCX)

S3 File (XLSX)

S4 File (XLSX)
