# Supplementary material for: Prevalence and associated factors of pediatric hypertension in Jazan region, south of the Kingdom of Saudi Arabia. A pilot cross-sectional study
Source: PLoS One. 2023 Jul 10;18(7):e0287698. doi: 10.1371/journal.pone.0287698 (PMC10332581; doi:10.1371/journal.pone.0287698)
Supplement: S1 File — (PDF) [file pone.0287698.s001.pdf]

Blood pressure measurement

Directions: How to apply your blood pressure cuff

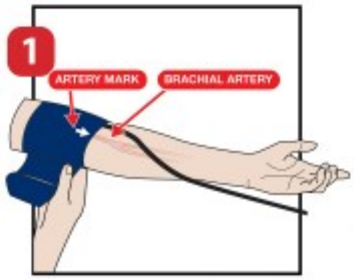

Step 1:

Secure the cuff on the left arm with the hook and loop fastener until the bottom edge is about ½" above your elbow. Align the artery mark and tubing to the brachial artery (inner arm).

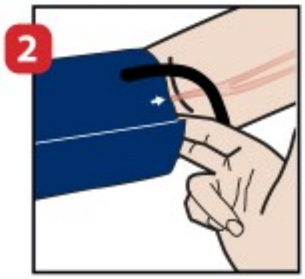

Step 2:

Tighten the cuff until in it snug against your arm, but not too tight. You should be able to fit 2 fingers between your arm and the cuff.

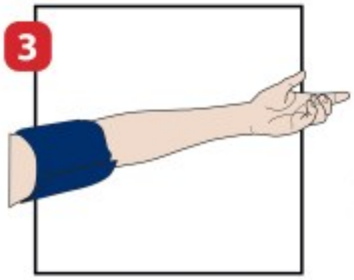

Step 3:

Sit and rest for 5 minutes prior to measurement. Relax your arm and follow the instructions that came with your blood pressure monitor.

② Keep the correct sitting position

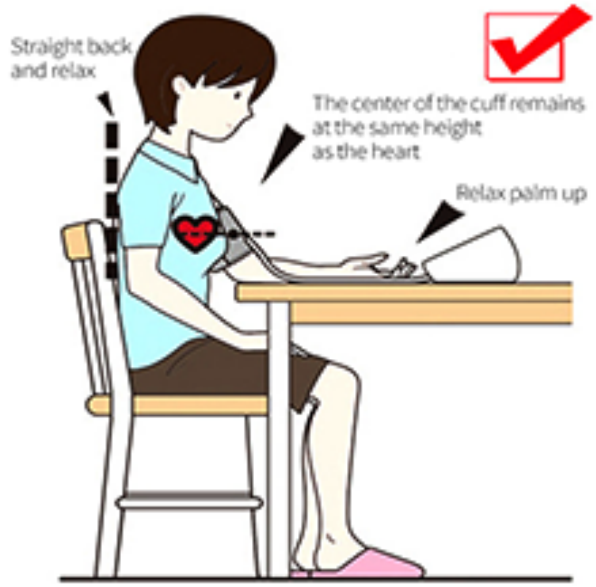

Note: Please sit in a room with a suitable temperature to measure your blood pressure, relax your body and sit naturally.

Please take at least 15 minutes of rest before measurement. For continuous measurement, please wait at least 2 minutes.

<Cuff wrap is too loose>

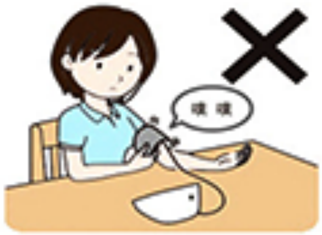

<Cuff wrap is too loose>

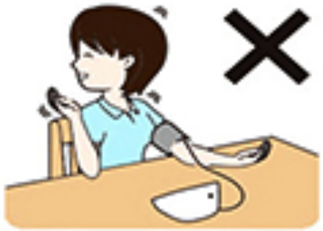

<Forward leaning posture>

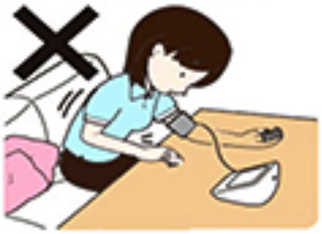

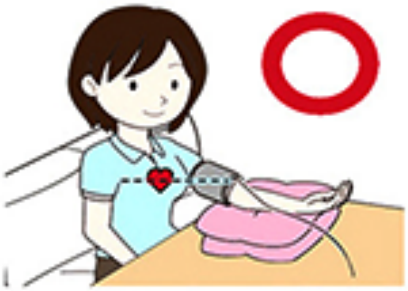

※ If the cuff is below the heart, place a cushion or towel underneath.

Cuff	Size	Limb Range Inches	Limb Range Centimeters
**Infant**	7	3.5 to 5.5	9 to 14
**Child**	9	5.1 to 7.6	13 to 19.5
**Small Adult**	10	7.4 to 10.6	19 to 27
**Adult**	11	9 to 15.7	23 to 40
**Large Adult**	12	13.3 to 19.6	34 to 50
**Thigh**	13	15.7 to 25.9	40 to 66

3 to 20 years: Boys
BP-for-stature percentiles

NAME ______________________________

RECORD # ____________________

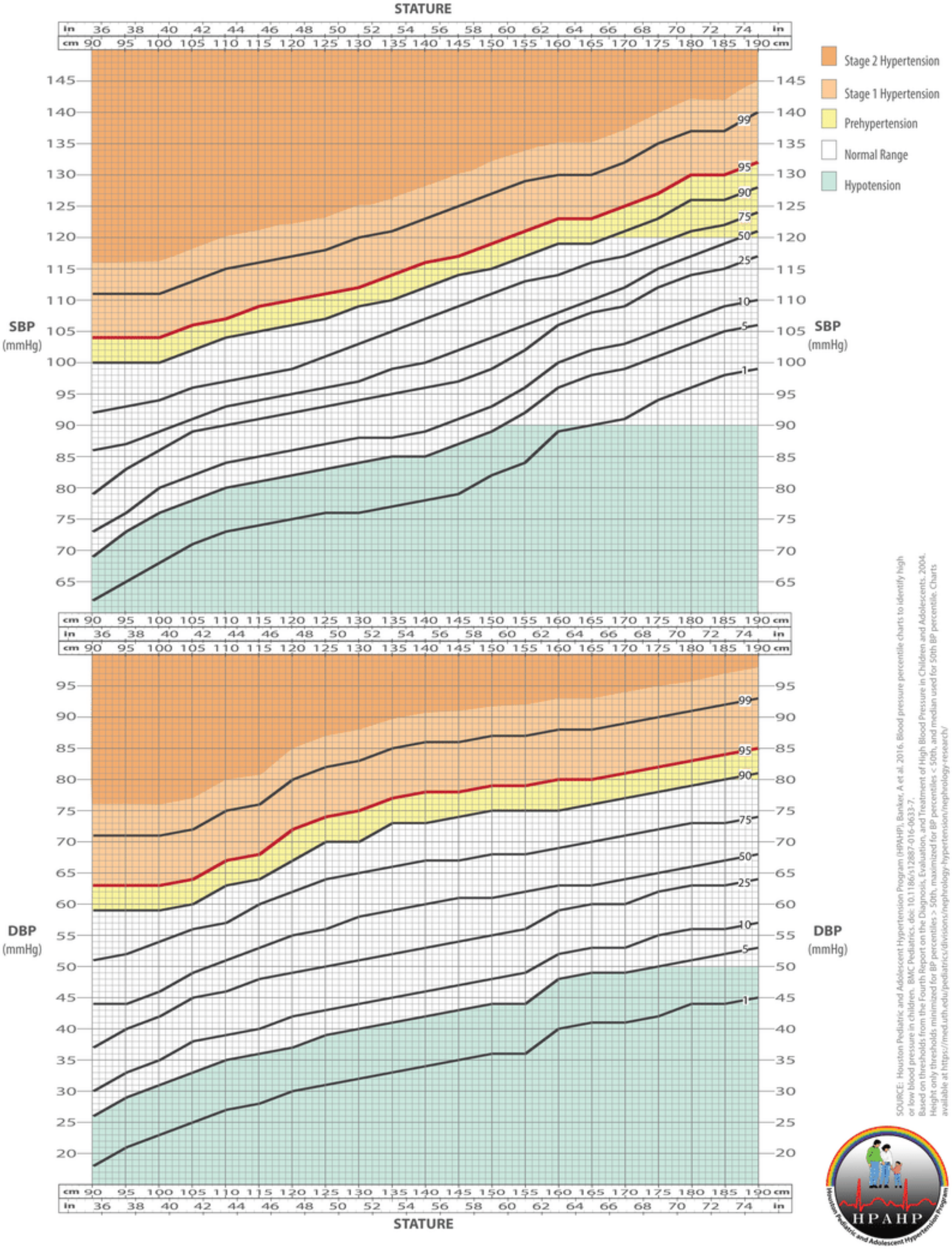

SOURCE: Houston Pediatric and Adolescent Hypertension Program (HPAHP), Banker, A et al. 2016. Blood pressure percentile charts to identify high or low blood pressure in children. BMC Pediatrics. doi: 10.1186/s12887-016-0633-7.
Based on thresholds from the Fourth Report on the Diagnosis, Evaluation, and Treatment of High Blood Pressure in Children and Adolescents. 2004.
Height only thresholds minimized for BP percentiles > 50th, maximized for BP percentiles < 50th, and median used for 50th BP percentile. Charts available at https://med.uth.edu/pediatrics/divisions/nephrology-hypertension/nephrology-research/

3 to 20 years: Girls
BP-for-stature percentiles

NAME ______________________

RECORD # ______________________

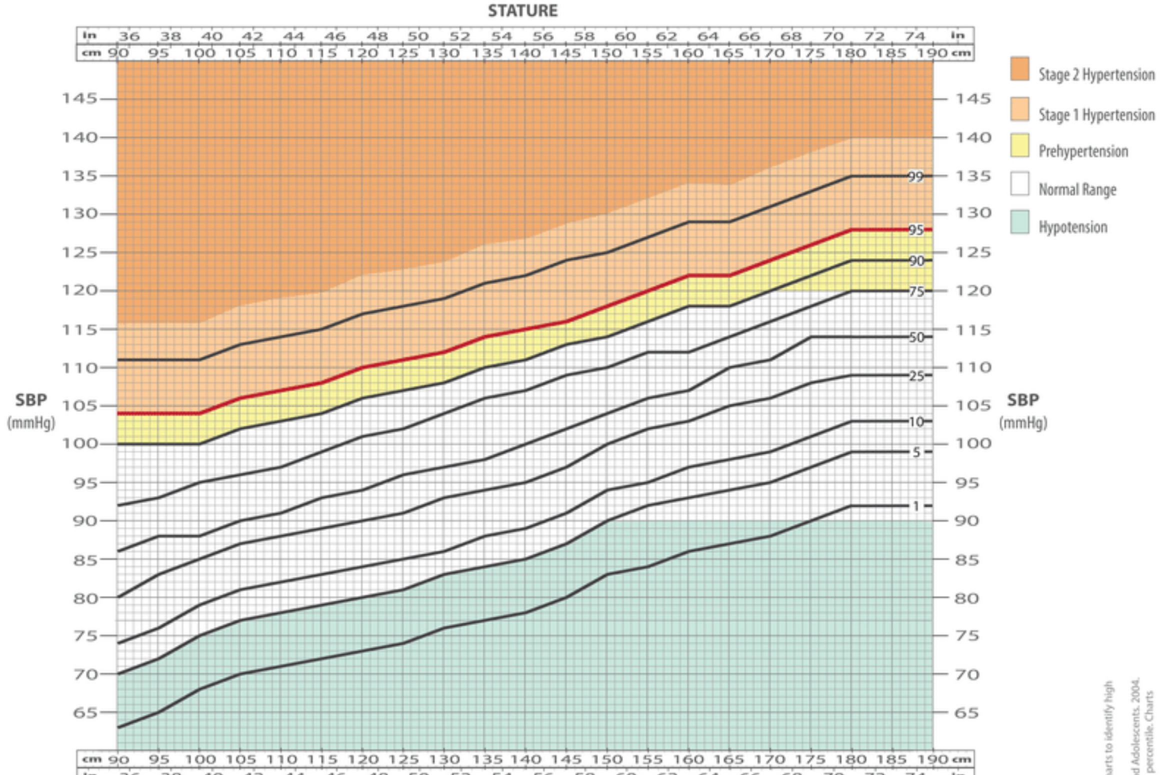

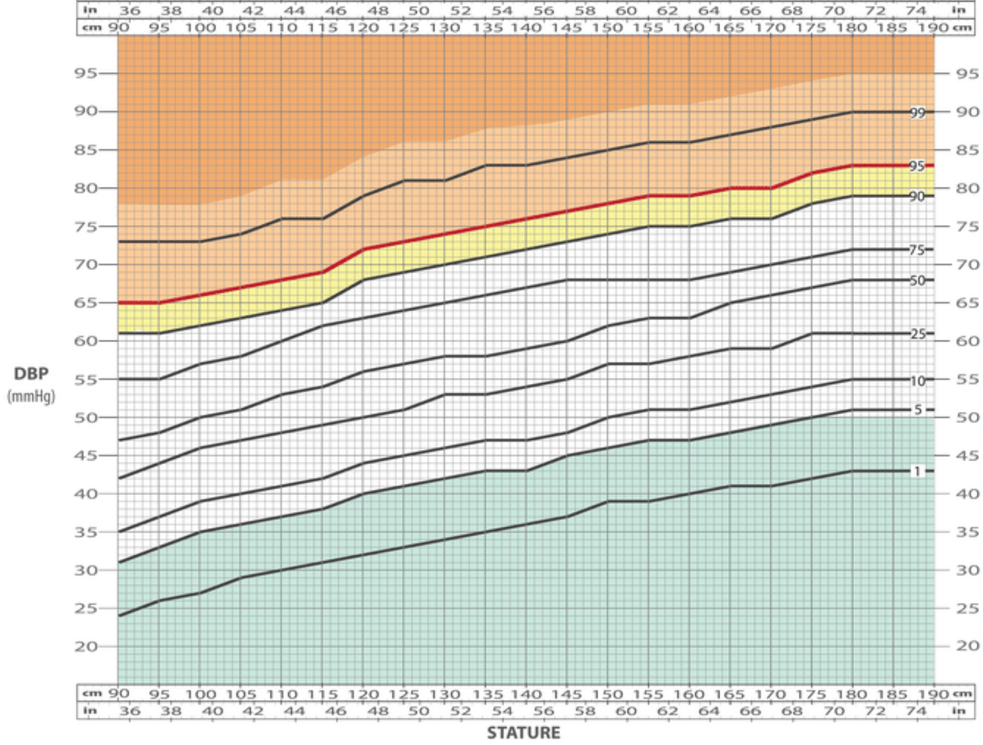

SOURCE: Houston Pediatric and Adolescent Hypertension Program (HPAHP), Banker, A et al. 2016. Blood pressure percentile charts to identify high or low blood pressure in children. BMC Pediatrics. doi: 10.1186/s12887-016-0633-7.
Based on thresholds from the Fourth Report on the Diagnosis, Evaluation, and Treatment of High Blood Pressure in Children and Adolescents. 2004.
Height only thresholds minimized for BP percentiles > 50th, maximized for BP percentiles < 50th, and median used for 50th BP percentile. Charts available at https://med.uth.edu/pediatrics/divisions/nephrology-hypertension/nephrology-research/

[illegible]

	2017 AAP CPG		2016 ESH Guidelines		2004 Fourth Report
	< 13 years	≥ 13 years	< 16 years	≥ 16 years	
Normal BP	< 90th percentile	< 120/< 80	< 90th percentile	< 130/85	< 90th percentile
Elevated BP*	≥ 90th to < 95th percentile or 120–129/< 80	120–129/< 80	≥ 90th to < 95th percentile	130–139/85–89	≥ 90th to < 95th percentile or > 120/80
Stage 1 HTN	≥ 95th to < 95th percentile + 12 mmHg or 130/80 to 139/89	130–139/80–89	≥ 95th to < 99th percentile + 5 mmHg	140–159/90–99	≥ 95th to < 99th percentile + 5 mmHg
Stage 2 HTN	≥ 95th percentile + 12 mmHg or ≥ 140/90	≥ 140/90	≥ 99th percentile + 5 mmHg	160–179/100–109	≥ 99th percentile + 5 mmHg

*Referred to as preHTN in the 2004 Fourth Report

Abbreviations: *AAP*, American Academy of Pediatrics; *BP*, blood pressure; *CPG*, clinical practice guideline; *ESH*, European Society of Hypertension; *HTN*, hypertension

3.2a. Simplified BP Table

This guideline includes a new, simplified table for initial BP screening (see Table 6) based on the 90th percentile BP for age and sex for children at the 5th percentile of height, which gives the values in the table a negative predictive value of >99%.[78] This simplified table is designed as a screening tool only for the identification of children and adolescents who need further evaluation of their BP starting with repeat BP measurements. It should not be used to diagnose elevated BP or HTN by itself. To diagnose elevated BP or HTN, it is important to locate the actual cutoffs in the complete BP tables because the SBP and DBP cutoffs may be as much as 9 mm Hg higher depending on a child's age and length or height. A typical-use case for this simplified table is for nursing staff to quickly identify BP that may need further evaluation by a clinician. For adolescents ≥13 years of age, a threshold of 120/80 mm Hg is used in the simplified table regardless of sex to align with adult guidelines for the detection of elevated BP.

https://publications.aap.org/pediatrics/article/140/3/e20171904/38358/Clinical-Practice-Guideline-for-Screening-and

TABLE 6

Screening BP Values Requiring Further Evaluation

Age, y	BP, mm Hg			
	Boys		Girls	
	Systolic	DBP	Systolic	DBP
1	98	52	98	54
2	100	55	101	58
3	101	58	102	60
4	102	60	103	62
5	103	63	104	64
6	105	66	105	67
7	106	68	106	68
8	107	69	107	69
9	107	70	108	71
10	108	72	109	72
11	110	74	111	74
12	113	75	114	75
≥13	120	80	120	80

BP Levels for Girls by Age and Height Percentile

Updated: January, 2012

Created by IPHA, 2011, all rights reserved

Age yrs	BP Percentile	Systolic BP (mmHg)							Diastolic BP (mmHg)						
		5th	10th	25th	50th	75th	90th	95th	5th	10th	25th	50th	75th	90th	95th
2	Height - *inches*	31.4	31.9	32.7	33.6	34.5	35.4	35.9	31.4	31.9	32.7	33.6	34.5	35.4	35.9
	Height - *cm*	79.6	80.9	83.0	85.4	87.7	89.9	91.1	79.6	80.9	83.0	85.4	87.7	89.9	91.1
	NT	85	85	87	88	89	91	91	43	44	44	45	46	46	47
	PreHT	98	99	100	101	103	104	105	57	58	58	59	60	61	61
	Stage 1 HT	102	103	104	105	107	108	109	61	62	62	63	64	65	65
	Stage 2 HT	114	115	116	117	119	120	121	74	74	75	75	76	77	77
3	Height - *inches*	34.6	35.1	36.0	37.1	38.1	39.1	39.7	34.6	35.1	36.0	37.1	38.1	39.1	39.7
	Height - *cm*	87.8	89.2	91.6	94.2	96.9	99.3	100.8	87.8	89.2	91.6	94.2	96.9	99.3	100.8
	NT	86	87	88	89	91	92	93	47	48	48	49	50	50	51
	PreHT	100	100	102	103	104	106	106	61	62	62	63	64	64	65
	Stage 1 HT	104	104	105	107	108	109	110	65	66	66	67	68	68	69
	Stage 2 HT	116	116	118	119	120	121	122	78	78	79	79	80	81	81
4	Height - *inches*	37.0	37.6	38.6	39.8	40.9	42.0	42.7	37.0	37.6	38.6	39.8	40.9	42.0	42.7
	Height - *cm*	94.0	95.6	98.1	101.0	104.0	106.8	108.4	94.0	95.6	98.1	101.0	104.0	106.8	108.4
	NT	88	88	90	91	92	94	94	50	50	51	52	52	53	54
	PreHT	101	102	103	104	106	107	108	64	64	65	66	67	67	68
	Stage 1 HT	105	106	107	108	110	111	112	68	68	69	70	71	71	72
	Stage 2 HT	117	118	119	120	122	123	124	81	81	81	82	83	84	84
5	Height - *inches*	39.5	40.2	41.3	42.5	43.8	45.0	45.7	39.5	40.2	41.3	42.5	43.8	45.0	45.7
	Height - *cm*	100.4	102.0	104.8	108.0	111.2	114.3	116.1	100.4	102.0	104.8	108.0	111.2	114.3	116.1
	NT	89	90	91	93	94	95	96	52	53	53	54	55	55	56
	PreHT	103	103	105	106	107	109	109	66	67	67	68	69	69	70
	Stage 1 HT	107	107	108	110	111	112	113	70	71	71	72	73	73	74
	Stage 2 HT	119	119	121	122	123	125	125	83	83	84	84	85	86	86
6	Height - *inches*	42.1	42.8	43.9	45.3	46.7	48.0	48.8	42.1	42.8	43.9	45.3	46.7	48.0	48.8
	Height - *cm*	106.9	108.6	111.6	115.0	118.6	121.9	123.9	106.9	108.6	111.6	115.0	118.6	121.9	123.9
	NT	91	92	93	94	96	97	98	54	54	55	56	56	57	58
	PreHT	104	105	106	108	109	110	111	68	68	69	70	70	71	72
	Stage 1 HT	108	109	110	111	113	114	115	72	72	73	74	74	75	76
	Stage 2 HT	120	121	122	124	125	126	127	85	85	85	86	87	88	88
7	Height - *inches*	44.5	45.2	46.5	47.9	49.4	50.8	51.7	44.5	45.2	46.5	47.9	49.4	50.8	51.7
	Height - *cm*	113.1	114.9	118.1	121.8	125.6	129.1	131.3	113.1	114.9	118.1	121.8	125.6	129.1	131.3
	NT	93	93	95	96	97	99	99	55	56	56	57	58	58	59
	PreHT	106	107	108	109	111	112	113	69	70	70	71	72	72	73
	Stage 1 HT	110	111	112	113	115	116	116	73	74	74	75	76	76	77
	Stage 2 HT	122	123	124	125	127	128	129	86	86	87	87	88	89	89
8	Height - *inches*	46.7	47.5	48.8	50.3	51.9	53.4	54.3	46.7	47.5	48.8	50.3	51.9	53.4	54.3
	Height - *cm*	118.5	120.5	123.9	127.8	131.9	135.6	137.9	118.5	120.5	123.9	127.8	131.9	135.6	137.9
	NT	95	95	96	98	99	100	101	57	57	57	58	59	60	60
	PreHT	108	109	110	111	113	114	114	71	71	71	72	73	74	74
	Stage 1 HT	112	112	114	115	116	118	118	75	75	75	76	77	78	78
	Stage 2 HT	124	125	126	127	128	130	130	87	87	88	88	89	90	91
9	Height - *inches*	48.5	19.3	50.8	52.4	54.1	55.7	56.6	48.5	19.3	50.8	52.4	54.1	55.7	56.6
	Height - *cm*	123.2	125.3	129.0	133.1	137.4	141.4	143.8	123.2	125.3	129.0	133.1	137.4	141.4	143.8
	NT	96	97	98	100	101	102	103	58	58	58	59	60	61	61
	PreHT	110	110	112	113	114	116	116	72	72	72	73	74	75	75
	Stage 1 HT	114	114	115	117	118	119	120	76	76	76	77	78	79	79
	Stage 2 HT	126	126	128	129	130	132	132	88	88	89	89	90	91	92

Age (y)	Blood Pressure Percentile	Systolic Blood Pressure (mmHg) Height Percentile or Measured Height							Diastolic Blood Pressure (mmHg) Height Percentile or Measured Height						
		5%	10%	25%	50%	75%	90%	95%	5%	10%	25%	50%	75%	90%	95%
10	Height (in)	51.1	52	53.7	55.5	57.4	59.1	60.2	51.1	52	53.7	55.5	57.4	59.1	60.2
	Height (cm)	129.7	132.2	136.3	141	145.8	150.2	152.8	129.7	132.2	136.3	141	145.8	150.2	152.8
	50th	96	97	98	99	101	102	103	58	59	59	60	61	61	62
	90th	109	110	111	112	113	115	116	72	73	73	73	73	73	73
	95th	113	114	114	116	117	119	120	75	75	76	76	76	76	76
	95th + 12 mmHg	125	126	126	128	129	131	132	87	87	88	88	88	88	88
11	Height (in)	53.4	54.5	56.2	58.2	60.2	61.9	63	53.4	54.5	56.2	58.2	60.2	61.9	63
	Height (cm)	135.6	138.3	142.8	147.8	152.8	157.3	160	135.6	138.3	142.8	147.8	152.8	157.3	160
	50th	98	99	101	102	104	105	106	60	60	60	61	62	63	64
	90th	111	112	113	114	116	118	120	74	74	74	74	74	75	75
	95th	115	116	117	118	120	123	124	76	77	77	77	77	77	77
	95th + 12 mmHg	127	128	129	130	132	135	136	88	89	89	89	89	89	89
12	Height (in)	56.2	57.3	59	60.9	62.8	64.5	65.5	56.2	57.3	59	60.9	62.8	64.5	65.5
	Height (cm)	142.8	145.5	149.9	154.8	159.6	163.8	166.4	142.8	145.5	149.9	154.8	159.6	163.8	166.4
	50th	102	102	104	105	107	108	108	61	61	61	62	64	65	65
	90th	114	115	116	118	120	122	122	75	75	75	75	76	76	76
	95th	118	119	120	122	124	125	126	78	78	78	78	79	79	79
	95th + 12 mmHg	130	131	132	134	136	137	138	90	90	90	90	91	91	91
13	Height (in)	58.3	59.3	60.9	62.7	64.5	66.1	67	58.3	59.3	60.9	62.7	64.5	66.1	67
	Height (cm)	148.1	150.6	154.7	159.2	163.7	167.8	170.2	148.1	150.6	154.7	159.2	163.7	167.8	170.2
	50th	104	105	106	107	108	108	109	62	62	63	64	65	65	66
	90th	116	117	119	121	122	123	123	75	75	75	76	76	76	76
	95th	121	122	123	124	126	126	127	79	79	79	79	80	80	81
	95th + 12 mmHg	133	134	135	136	138	138	139	91	91	91	91	92	92	93
14	Height (in)	59.3	60.2	61.8	63.5	65.2	66.8	67.7	59.3	60.2	61.8	63.5	65.2	66.8	67.7
	Height (cm)	150.6	153	156.9	161.3	165.7	169.7	172.1	150.6	153	156.9	161.3	165.7	169.7	172.1
	50th	105	106	107	108	109	109	109	63	63	64	65	66	66	66
	90th	118	118	120	122	123	123	123	76	76	76	76	77	77	77
	95th	123	123	124	125	126	127	127	80	80	80	80	81	81	82
	95th + 12 mmHg	135	135	136	137	138	139	139	92	92	92	92	93	93	94
15	Height (in)	59.7	60.6	62.2	63.9	65.6	67.2	68.1	59.7	60.6	62.2	63.9	65.6	67.2	68.1
	Height (cm)	151.7	154	157.9	162.3	166.7	170.6	173	151.7	154	157.9	162.3	166.7	170.6	173
	50th	105	106	107	108	109	109	109	64	64	64	65	66	67	67
	90th	118	119	121	122	123	123	124	76	76	76	77	77	78	78
	95th	124	124	125	126	127	127	128	80	80	80	81	82	82	82
	95th + 12 mmHg	136	136	137	138	139	139	140	92	92	92	93	94	94	94
16	Height (in)	59.9	60.8	62.4	64.1	65.8	67.3	68.3	59.9	60.8	62.4	64.1	65.8	67.3	68.3
	Height (cm)	152.1	154.5	158.4	162.8	167.1	171.1	173.4	152.1	154.5	158.4	162.8	167.1	171.1	173.4
	50th	106	107	108	109	109	110	110	64	64	65	66	66	67	67
	90th	119	120	122	123	124	124	124	76	76	76	77	78	78	78
	95th	124	125	125	127	127	128	128	80	80	80	81	82	82	82
	95th + 12 mmHg	136	137	137	139	139	140	140	92	92	92	93	94	94	94
17	Height (in)	60.0	60.9	62.5	64.2	65.9	67.4	68.4	60.0	60.9	62.5	64.2	65.9	67.4	68.4
	Height (cm)	152.4	154.7	158.7	163.0	167.4	171.3	173.7	152.4	154.7	158.7	163.0	167.4	171.3	173.7
	50th	107	108	109	110	110	110	111	64	64	65	66	66	66	67
	90th	120	121	123	124	124	125	125	76	76	77	77	78	78	78
	95th	125	125	126	127	128	128	128	80	80	80	81	82	82	82
	95th + 12 mmHg	137	137	138	139	140	140	140	92	92	92	93	94	94	94

A Boys

Age (y)	Blood Pressure Percentile	Systolic Blood Pressure (mmHg) Height Percentile or Measured Height							Diastolic Blood Pressure (mmHg) Height Percentile or Measured Height						
		5%	10%	25%	50%	75%	90%	95%	5%	10%	25%	50%	75%	90%	95%
1	Height (in)	30.4	30.8	31.6	32.4	33.3	34.1	34.6	30.4	30.8	31.6	32.4	33.3	34.1	34.6
	Height (cm)	77.2	78.3	80.2	82.4	84.6	86.7	87.9	77.2	78.3	80.2	82.4	84.6	86.7	87.9
	50th	85	85	86	86	87	88	88	40	40	40	41	41	42	42
	90th	98	99	99	100	100	101	101	52	52	53	53	54	54	54
	95th	102	102	103	103	104	105	105	54	54	55	55	56	57	57
	95th + 12 mmHg	114	114	115	115	116	117	117	66	66	67	67	68	69	69
2	Height (in)	33.9	34.4	35.3	36.3	37.3	38.2	38.8	33.9	34.4	35.3	36.3	37.3	38.2	38.8
	Height (cm)	86.1	87.4	89.6	92.1	94.7	97.1	98.5	86.1	87.4	89.6	92.1	94.7	97.1	98.5
	50th	87	87	88	89	89	90	91	43	43	44	44	45	46	46
	90th	100	100	101	102	103	103	104	55	55	56	56	57	58	58
	95th	104	105	105	106	107	107	108	57	58	58	59	60	61	61
	95th + 12 mmHg	116	117	117	118	119	119	120	69	70	70	71	72	73	73
3	Height (in)	36.4	37	37.9	39	40.1	41.1	41.7	36.4	37	37.9	39	40.1	41.1	41.7
	Height (cm)	92.5	93.9	96.3	99	101.8	104.3	105.8	92.5	93.9	96.3	99	101.8	104.3	105.8
	50th	88	89	89	90	91	92	92	45	46	46	47	48	49	49
	90th	101	102	102	103	104	105	105	58	58	59	59	60	61	61
	95th	106	106	107	107	108	109	109	60	61	61	62	63	64	64
	95th + 12 mmHg	118	118	119	119	120	121	121	72	73	73	74	75	76	76
4	Height (in)	38.8	39.4	40.5	41.7	42.9	43.9	44.5	38.8	39.4	40.5	41.7	42.9	43.9	44.5
	Height (cm)	98.5	100.2	102.9	105.9	108.9	111.5	113.2	98.5	100.2	102.9	105.9	108.9	111.5	113.2
	50th	90	90	91	92	93	94	94	48	49	49	50	51	52	52
	90th	102	103	104	105	105	106	107	60	61	62	62	63	64	64
	95th	107	107	108	108	109	110	110	63	64	65	66	67	67	68
	95th + 12 mmHg	119	119	120	120	121	122	122	75	76	77	78	79	79	80
5	Height (in)	41.1	41.8	43.0	44.3	45.5	46.7	47.4	41.1	41.8	43.0	44.3	45.5	46.7	47.4
	Height (cm)	104.4	106.2	109.1	112.4	115.7	118.6	120.3	104.4	106.2	109.1	112.4	115.7	118.6	120.3
	50th	91	92	93	94	95	96	96	51	51	52	53	54	55	55
	90th	103	104	105	106	107	108	108	63	64	65	65	66	67	67
	95th	107	108	109	109	110	111	112	66	67	68	69	70	70	71
	95th + 12 mmHg	119	120	121	121	122	123	124	78	79	80	81	82	82	83
6	Height (in)	43.4	44.2	45.4	46.8	48.2	49.4	50.2	43.4	44.2	45.4	46.8	48.2	49.4	50.2
	Height (cm)	110.3	112.2	115.3	118.9	122.4	125.6	127.5	110.3	112.2	115.3	118.9	122.4	125.6	127.5
	50th	93	93	94	95	96	97	98	54	54	55	56	57	57	58
	90th	105	105	106	107	109	110	110	66	66	67	68	68	69	69
	95th	108	109	110	111	112	113	114	69	70	70	71	72	72	73
	95th + 12 mmHg	120	121	122	123	124	125	126	81	82	82	83	84	84	85
7	Height (in)	45.7	46.5	47.8	49.3	50.8	52.1	52.9	45.7	46.5	47.8	49.3	50.8	52.1	52.9
	Height (cm)	116.1	118	121.4	125.1	128.9	132.4	134.5	116.1	118	121.4	125.1	128.9	132.4	134.5
	50th	94	94	95	97	98	98	99	56	56	57	58	58	59	59
	90th	106	107	108	109	110	111	111	68	68	69	70	70	71	71
	95th	110	110	111	112	114	115	116	71	71	72	73	73	74	74
	95th + 12 mmHg	122	122	123	124	126	127	128	83	83	84	85	85	86	86
8	Height (in)	47.8	48.6	50	51.6	53.2	54.6	55.5	47.8	48.6	50	51.6	53.2	54.6	55.5
	Height (cm)	121.4	123.5	127	131	135.1	138.8	141	121.4	123.5	127	131	135.1	138.8	141
	50th	95	96	97	98	99	99	100	57	57	58	59	59	60	60
	90th	107	108	109	110	111	112	112	69	70	70	71	72	72	73
	95th	111	112	112	114	115	116	117	72	73	73	74	75	75	75
	95th + 12 mmHg	123	124	124	126	127	128	129	84	85	85	86	87	87	87
9	Height (in)	49.6	50.5	52	53.7	55.4	56.9	57.9	49.6	50.5	52	53.7	55.4	56.9	57.9
	Height (cm)	126	128.3	132.1	136.3	140.7	144.7	147.1	126	128.3	132.1	136.3	140.7	144.7	147.1
	50th	96	97	98	99	100	101	101	57	58	59	60	61	62	62
	90th	107	108	109	110	112	113	114	70	71	72	73	74	74	74
	95th	112	112	113	115	116	118	119	74	74	75	76	76	77	77
	95th + 12 mmHg	124	124	125	127	128	130	131	86	86	87	88	88	89	89

Age (y)	Blood Pressure Percentile	Systolic Blood Pressure (mmHg)							Diastolic Blood Pressure (mmHg)						
		Height Percentile or Measured Height							Height Percentile or Measured Height						
		5%	10%	25%	50%	75%	90%	95%	5%	10%	25%	50%	75%	90%	95%
10	Height (in)	51.3	52.2	53.8	55.6	57.4	59.1	60.1	51.3	52.2	53.8	55.6	57.4	59.1	60.1
	Height (cm)	130.2	132.7	136.7	141.3	145.9	150.1	152.7	130.2	132.7	136.7	141.3	145.9	150.1	152.7
	50th	97	98	99	100	101	102	103	59	60	61	62	63	63	64
	90th	108	109	111	112	113	115	116	72	73	74	74	75	75	76
	95th	112	113	114	116	118	120	121	76	76	77	77	78	78	78
	95th + 12 mmHg	124	125	126	128	130	132	133	88	88	89	89	90	90	90
11	Height (in)	53	54	55.7	57.6	59.6	61.3	62.4	53	54	55.7	57.6	59.6	61.3	62.4
	Height (cm)	134.7	137.3	141.5	146.4	151.3	155.8	158.6	134.7	137.3	141.5	146.4	151.3	155.8	158.6
	50th	99	99	101	102	103	104	106	61	61	62	63	63	63	63
	90th	110	111	112	114	116	117	118	74	74	75	75	75	76	76
	95th	114	114	116	118	120	123	124	77	78	78	78	78	78	78
	95th + 12 mmHg	126	126	128	130	132	135	136	89	90	90	90	90	90	90
12	Height (in)	55.2	56.3	58.1	60.1	62.2	64	65.2	55.2	56.3	58.1	60.1	62.2	64	65.2
	Height (cm)	140.3	143	147.5	152.7	157.9	162.6	165.5	140.3	143	147.5	152.7	157.9	162.6	165.5
	50th	101	101	102	104	106	108	109	61	62	62	62	62	63	63
	90th	113	114	115	117	119	121	122	75	75	75	75	75	76	76
	95th	116	117	118	121	124	126	128	78	78	78	78	78	79	79
	95th + 12 mmHg	128	129	130	133	136	138	140	90	90	90	90	90	91	91
13	Height (in)	57.9	59.1	61	63.1	65.2	67.1	68.3	57.9	59.1	61	63.1	65.2	67.1	68.3
	Height (cm)	147	150	154.9	160.3	165.7	170.5	173.4	147	150	154.9	160.3	165.7	170.5	173.4
	50th	103	104	105	108	110	111	112	61	60	61	62	63	64	65
	90th	115	116	118	121	124	126	126	74	74	74	75	76	77	77
	95th	119	120	122	125	128	130	131	78	78	78	78	80	81	81
	95th + 12 mmHg	131	132	134	137	140	142	143	90	90	90	90	92	93	93
14	Height (in)	60.6	61.8	63.8	65.9	68.0	69.8	70.9	60.6	61.8	63.8	65.9	68.0	69.8	70.9
	Height (cm)	153.8	156.9	162	167.5	172.7	177.4	180.1	153.8	156.9	162	167.5	172.7	177.4	180.1
	50th	105	106	109	111	112	113	113	60	60	62	64	65	66	67
	90th	119	120	123	126	127	128	129	74	74	75	77	78	79	80
	95th	123	125	127	130	132	133	134	77	78	79	81	82	83	84
	95th + 12 mmHg	135	137	139	142	144	145	146	89	90	91	93	94	95	96
15	Height (in)	62.6	63.8	65.7	67.8	69.8	71.5	72.5	62.6	63.8	65.7	67.8	69.8	71.5	72.5
	Height (cm)	159	162	166.9	172.2	177.2	181.6	184.2	159	162	166.9	172.2	177.2	181.6	184.2
	50th	108	110	112	113	114	114	114	61	62	64	65	66	67	68
	90th	123	124	126	128	129	130	130	75	76	78	79	80	81	81
	95th	127	129	131	132	134	135	135	78	79	81	83	84	85	85
	95th + 12 mmHg	139	141	143	144	146	147	147	90	91	93	95	96	97	97
16	Height (in)	63.8	64.9	66.8	68.8	70.7	72.4	73.4	63.8	64.9	66.8	68.8	70.7	72.4	73.4
	Height (cm)	162.1	165	169.6	174.6	179.5	183.8	186.4	162.1	165	169.6	174.6	179.5	183.8	186.4
	50th	111	112	114	115	115	116	116	63	64	66	67	68	69	69
	90th	126	127	128	129	131	131	132	77	78	79	80	81	82	82
	95th	130	131	133	134	135	136	137	80	81	83	84	85	86	86
	95th + 12 mmHg	142	143	145	146	147	148	149	92	93	95	96	97	98	98
17	Height (in)	64.5	65.5	67.3	69.2	71.1	72.8	73.8	64.5	65.5	67.3	69.2	71.1	72.8	73.8
	Height (cm)	163.8	166.5	170.9	175.8	180.7	184.9	187.5	163.8	166.5	170.9	175.8	180.7	184.9	187.5
	50th	114	115	116	117	117	118	118	65	66	67	68	69	70	70
	90th	128	129	130	131	132	133	134	78	79	80	81	82	82	83
	95th	132	133	134	135	137	138	138	81	82	84	85	86	86	87
	95th + 12 mmHg	144	145	146	147	149	150	150	93	94	96	97	98	98	99
