# Supplementary material for: Prevalence and associated factors of pediatric hypertension in Jazan region, south of the Kingdom of Saudi Arabia. A pilot cross-sectional study
Source: PLoS One. 2023 Jul 10;18(7):e0287698. doi: 10.1371/journal.pone.0287698 (PMC10332581; doi:10.1371/journal.pone.0287698)
Supplement: S2 File — (DOCX) [file pone.0287698.s002.docx]

| Coding | Variable |
| --- | --- |
| 1=male  2= female | Gender |
| 1= city  2= village  3=mountains | Residence |
| 0= No  1= yes | Are you in school? |
| 1= kindergarten  2= first grade of primary school  3= second grade of primary school  4= thir grade of primary school  5= fourth grade of primary school  6= fifth grade of primary school  7= sixth grade of primary school  8= first grade of preparatory school  9= second grade of preparatory school  10= third grade of preparatory school  11= first grade of secondary school  12= Don’t study | In which grade? |
| 1= Didn’t study  2= Primary school  3= preparatory school  4= secondary school or diploma  5= Bachelor  6= Master or Doctorate | Father's Education |
| 1= non-working  2= employed  3=soldier  4= self- employed | What is his job |
| 1= Didn’t study  2= Primary school  3= preparatory school  4= secondary school or diploma  5= Bachelor  6= Master or Doctorate | Mother's Education |
| 1= non-working  2= employed  3=soldier  4= self- employed | What is her job |
| 0= No  1= yes | Consanguinity |
| 0= No  1= yes | Do you have siblings? |
| 1= oldest  2= middle  3= youngest | What is your order among them? |
| 1= 0-5000 SR  2= 5000-10000 SR  3= 10000-15000 SR  4= above 15000 SR | Family Income |
| 0= No  1= yes | Do you have family history of HTN? |
| 1= father  2= mother  3= grandfather  4= grandmother  5= father & mother  6= grandfather & grandmother  7= all family members | Who is the affected one? |
| 0= No  1= yes | Do you suffer from any medical Condition? |
| 1= Bronchial asthma  2= diabetes  3= epilepsy  4= Anemia  5=G6PD Deficiency Anemia  6= Ventricular septal defect  7= Eczema  8= Leukemia  9=Nephromegaly | What it is? |
| 1= Normotinsive  2= PreHypertinsive  3= Hypertinsive | Diagnosis |
| 1= underwieght  2= Normal body wieght  3= Overwight  4= obese | BMI diagnosis |
